# Olfactory Ecto-Mesenchymal Stem Cell-Derived Exosomes Ameliorate Experimental Colitis *via* Modulating Th1/Th17 and Treg Cell Responses

**DOI:** 10.3389/fimmu.2020.598322

**Published:** 2020-12-10

**Authors:** Jie Tian, Qiugang Zhu, Yidan Zhang, Qianying Bian, Yue Hong, Ziwei Shen, Huaxi Xu, Ke Rui, Kai Yin, Shengjun Wang

**Affiliations:** ^1^ Department of Laboratory Medicine, The Affiliated People’s Hospital, Jiangsu University, Zhenjiang, China; ^2^ Department of Immunology, Jiangsu Key Laboratory of Laboratory Medicine, School of Medicine, Jiangsu University, Zhenjiang, China; ^3^ Department of Laboratory Medicine, Affiliated Hospital of Jiangsu University, Zhenjiang, China; ^4^ Department of General Surgery, Affiliated Hospital of Jiangsu University, Zhenjiang, China

**Keywords:** exosome, inflammatory bowel disease, immunoregulation, T cells, olfactory ecto-mesenchymal stem cells

## Abstract

Olfactory ecto-mesenchymal stem cells (OE-MSCs) are a novel population of resident stem cells in the olfactory lamina propria with strong immunosuppressive function. Exosomes released by MSCs are considered to carry various mRNAs, microRNAs and proteins from cells and function as an extension of MSCs. However, it remains unclear whether exosomes derived from OE-MSCs (OE-MSCs-Exos) possess any immunoregulatory functions. In this study, we found that OE-MSCs-Exos possessed strong suppressive function in CD4^+^T cell proliferation, accompanied by reduced IL-17, IFN-γ and enhanced TGF-β, IL-10 secreted by T cells. In experimental colitis mice, treatment of OE-MSCs-Exos markedly alleviated the severity of disease, and Th1/Th17 subpopulations were remarkably reduced whereas Treg cells were increased after OE-MSCs-Exos treatment. Mechanistically, OE-MSCs-Exos were demonstrated to inhibit the differentiation of Th1 and Th17 cells, but promote the induction of Treg cells *in vitro*. Taken together, our findings identified a novel function of OE-MSCs-Exos in regulating T-cell responses, indicating that OE-MSCs-Exos may represent a new cell-free therapy for the treatment of IBD and other inflammatory diseases.

## Introduction

Inflammatory bowel disease (IBD) is a chronic, relapsing, and remitting inflammatory disorder of intestinal tract, including Crohn’s disease and ulcerative colitis. The incidence of IBD has increased worldwide and challenges the public health (1). It has been acknowledged that the long-term chronic inflammation in the intestine can cause abscesses, fistulas, extraintestinal manifestations, and even colitis-associated cancer (2). Currently, the etiology of IBD is associated with the genetics, gut microbiota, immune responses, and the environmental factors (3). The aberrant immune responses are demonstrated to be closely associated with the chronic inflammation in IBD. Accumulated studies have implied multiple adaptive immune cells are involved in the pathogenesis of IBD, including Th1, Th17 and regulatory T cells (Tregs) (4). To date, those traditional drugs, such as antibiotics, corticosteroids, and immunosuppressive agents, can only offer temporary remission but may result in the several side effects, including psoriasis, hypersensitivity, and drug-induced cytotoxicity (2). Thereafter, exploring novel alternative therapies for IBD is necessary.

Mesenchymal stem cells (MSCs) is a population of stromal cells with the capacity of self-renewal and multipotent differentiation potential. The immunomodulatory and anti-inflammatory properties of MSCs have provided powerful competition for MSCs in the cellular-based therapy for a variety of immune-mediated disorders, including autoimmune diseases and inflammatory diseases (5). In recent years, MSC-based therapeutic intervention has emerged as a promising strategy for the treatment of IBD in clinic trails (6, 7). However, the transplantation of MSCs has some shortcomings, such as the unstable phenotype from the different batches, cellular rejection, high costs in storage, transport, and recovery. Additionally, the transplanted MSCs can be modified *in vivo* by the complicated environment, leading to the invalid or unwanted effect in patients (8). Considering these drawbacks in the cell-based therapy, a novel cell-free therapy, MSC-secreted extracellular vesicles (EVs), including microvesicles and exosomes, have recently emerged as a powerful tool in the treatment of various diseases, such as degenerative diseases, graft-versus-host diseases, and inflammatory diseases (9–12). Exosomes are nano-sized EVs (40–100 nm in diameter) that possess remarkable physiological properties and originate *via* the inward budding of the membrane of late endosomes (13). Current researches indicate that exosomes derived from MSCs (MSC-Exos) mediate cell-cell micro-communication and transport paracrine factors during tissue repair and immune regulation, thus efficiently enhancing the therapeutic potency of MSCs in several diseases (14–18).

Olfactory ecto-mesenchymal stem cells (OE-MSCs) is a new type of stem cells resident in the olfactory lamina propria. As a population of stem cells, OE-MSCs possess the capability of self-renewal, high proliferation rate and multiple lineage differentiation potential (19, 20). Our previous data have identified that OE-MSCs exhibited powerful immunomodulation capacity and ameliorated the severity of murine collagen-induced arthritis *via* regulating T cell responses (21). However, our exploration further showed that the immunosuppressive function of OE-MSCs could be down-regulated by IL-17, indicating the endogenous inflammatory microenvironment could modify the transplanted cells and impair the therapeutic potential of OE-MSCs in clinical application (22). Therefore, as a novel cell-free therapy, OE-MSCs-derived exosomes (OE-MSCs-Exos) are supposed to provide multiple advantages over cell-based treatment, and might possess great therapeutic potential in inflammatory diseases.

In this study, we investigated the immunomodulatory function of OE-MSCs-Exos in regulating T-cell responses. We found that OE-MSCs-Exos exerted their immunosuppressive capacity *via* inhibiting Th1 and Th17 cell differentiation and promoting regulatory T (Treg) cell induction. By using the experimental colitis mouse model, OE-MSCs-Exos were demonstrated to alleviate the disease severity, accompanied by decreased Th1/Th17 cell responses and increased Treg cells *in vivo*. Thus, our findings identified the capacity of OE-MSCs-Exos in regulating T-cell functions, indicating that OE-MSCs-Exos may present an effective therapeutic tool for the treatment of IBD.

## Materials and Methods

### Mice

Male C57BL/6 mice at 6–8-week old were purchased from Experimental Animal Center of Yangzhou University. Mice were housed in a specific pathogen-free animal facility, and all the experiments were approved by the Committee on the Use of Live Animals in Research and Teaching of Jiangsu University.

### Isolation and Culture of OE-MSCs

OE-MSCs were isolated from the nasal cavity of C57BL/6 mice as we previously described (21). Briefly, the olfactory epithelium tissue was cut into small pieces and cultured in flasks with the medium DMEM/F-12 supplemented with 15% fetal calf serum) (Gibco) for 7 days. After removing the non-adherent cells, the remaining cells were trypsinized and further expanded for 3 passages.

### Osteogenic and Adipogenic Differentiation Assay

Osteogenic and adipogenic differentiation of OE-MSCs was analyzed as we previously described (21). For osteogenic induction, OE-MSCs were cultured in osteogenic induction medium for 3 weeks, and then the osteogenic differentiation was detected by alizarin red (Cyagen) staining. For adipogenic differentiation, OE-MSCs were cultured in adipogenic differentiation medium (Cyagen) for 14 days, and then cells were stained with oil red O (Cyagen).

### Isolation of OE-MSCs-Exos

OE-MSCs were cultured in the medium with exosomes-depleted FBS for 48 h, and the supernatants were collected to extract exosomes by ultracentrifuged method. Exosomes were purified from the supernatants by differential centrifugation to remove cells and debris, and then the supernatants were ultracentrifuged at 100,000 g for 60 min at 4°C. After removing the supernatants, the exosomal pellets were washed in PBS and centrifuged at 100,000 g for another 60 min at 4°C, and then resuspended in PBS and stored at -80°C. The protein concentration of OE-MSCs-Exos was quantified using a micro-BCA protein assay kit.

### Electron Microscopy

The transmission electron microscopy (TEM) analysis was performed as we previously described (23). Briefly, OE-MSCs-Exos suspension were fixed in 4% paraformaldehyde at 4°C for 60 min. Then, the exosomal pellets were applied to a formvar-coated grid and negatively stained with 3% aqueous phosphotungstic acid. The TEM sample imaging was performed using transmission electron microscopy (Tecnai-12, Philips).

For scanning electron microscopy (SEM), exosomes were fixed with 3.7% glutaraldehyde and then dehydrated with ethanol. Sections were left to dry at room temperature for 24 h and then analyzed by SEM.

### Nanoparticle Tracking Analysis

The sizes of OE-MSCs-Exos were measured by NTA using a ZetaView PMX 110 (Particle Metrix) and analyzed by the software ZetaView 8.04.02.

### Western Blotting Analysis

Proteins extracted from the exosomes were separated by 12% SDS-PAGE, then transferred onto Immobilon polyvinylidene membranes (Bio-Rad) and probed with antibodies against CD9, CD63 and calnexin (Abcam) followed by chemiluminescent detection (Champion Chemical).

### Flow Cytometric Analysis

For surface markers, MSCs were collected to stain with relevant fluorochrome-conjugated monoclonal antibodies (mAbs): anti-mouse CD29, CD90, CD44, CD34, CD45, and CD11b from eBioscience. Lymph nodes were isolated from mice and ground in culture medium. The suspensions were filtered through 70-μm cell strainers. For intracellular cytokine staining, single cell suspensions were stimulated with PMA (Sigma-Aldrich, 50 ng/ml), ionomycin (Enzo, 1 μg/ml), and monensin (Enzo, 2 μg/ml). After 5 h, cells were stained with anti-CD4 mAb (eBioscience), fixed, permeabilized, and stained with anti-IFN-γ or IL-17 mAbs (eBioscience) according to the Intracellular Staining Kit (Invitrogen, Carlsbad, CA) instructions. For Treg cells staining, anti-CD4, anti-CD25, and anti-Foxp3 mAbs (eBioscience) were performed following Foxp3 Staining Buffer Set (eBioscience) protocols. Flow cytometry was performed using the BD FACSCanto II (Becton Dickinson) and data were analyzed using FlowJo software (Becton Dickinson) (24).

### T Cell Suppression Assay

Mouse CD4^+^ T cells were sorted from wild-type mice using CD4^+^T cell microbeads (Miltenyi Biotec), and the purity of CD4^+^T cells was >95%. The isolated CD4^+^ T cells labeled with carboxyfluorescein succinimidyl ester (CFSE, 5 mM; Invitrogen) were treated with or without OE-MSCs-Exos in the presence of anti-CD3 and anti-CD28 mAbs (eBioscience) for 72 h. CFSE fluorescence intensity was analyzed to determine the proliferation of CD4^+^ T cells by flow cytometry.

### Enzyme-Linked Immunosorbent Assay

Concentrations IFN-γ, IL-17, TGF-β, and IL-10 in the supernatants were detected by sandwich ELISA (eBioscience). All Assays were performed according to the manufacturer’s instructions. Briefly, samples were incubated in 96-well plates pre-coated with the capture antibody for 2 h at room temperature, followed by incubation of biotin-conjugated detection antibody for 1 h. After washing, HRP Streptavidin was added and incubated for 30 min. Then, plates were washed and the TMB substrate was added. After 30 min, stop solution was added and absorbance was measured at 450 nm using a microplate reader (Biotek Winooski).

### DSS-Induced Experimental Colitis

Male C57BL/6 mice were subjected to 2.5% DSS treatment from day 0 to day 4, followed by water treatment from day 4 to 7 to induce the colitis. Mice were intravenously administered with PBS or exosomes (60 µg/mouse) on days 2 and 4 after DSS drinking. Weight loss, stool consistency and rectal bleeding were monitored daily for 7 days and scored separately on scales of 0–4. The disease activity index (DAI) was calculated according to the average values of the three values. Colons were isolated and the lengths were measured. For the histological assessment, colons were fixed in 10% formalin solution, paraffin-embedded, sections and then stained with hematoxylin and eosin (H & E), the histological scoring was determined with a combined score for tissue damage (score, 0–3) and inflammatory cell infiltration (score, 0–3) as described previously (25).

### Induction of T Cell Subsets

Naïve CD4^+^ T cells were isolated using CD4^+^CD62L^+^T Cell Isolation Kit II (Miltenyi Biotec). Naïve CD4^+^ T cells were cultured in a 24-well plate precoated with anti-CD3 (1 μg/ml) and anti-CD28 (1 μg/ml) mAbs under Th1, Th17, and Treg cell induction conditions for 3 days. Cytokines for T cell subset differentiation are as follows: Th1, IL-12 (5 ng/ml) and anti-IL-4 mAb (10 μg/ml); Th17, TGF-β (3 ng/ml), IL-6 (20 ng/ml), IL-23 (20 ng/ml), anti-IL-4 mAb (10 μg/ml) and IFN-γ mAb (10 μg/ml); Treg, TGF-β (2.5 ng/ml).

### Statistical Analysis

The statistical significance was determined by the Student’s t test or one-way, ANOVA. All analyses were performed using SPSS 16.0 software. P values <0.05 were considered statistically significant.

## Results

### Characterization of OE-MSCs-Secreted Exosomes

**Figure 1:**
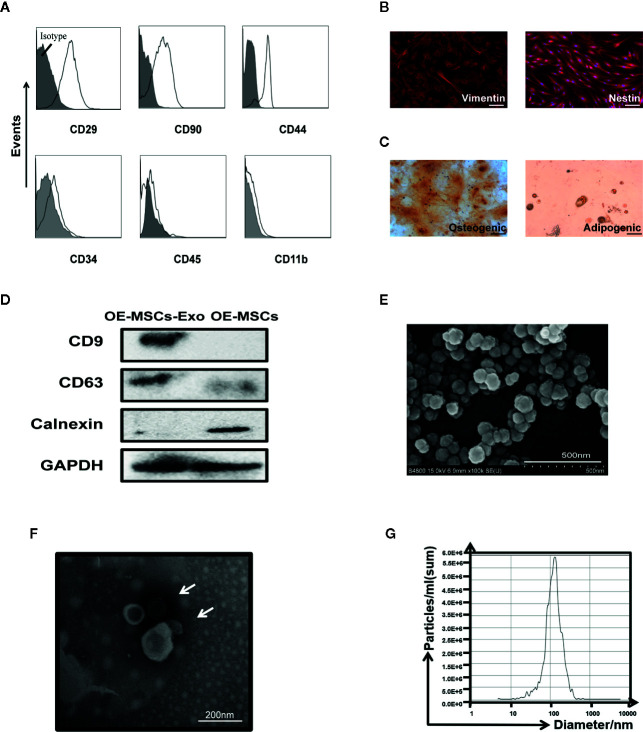
Identification of OE-MSCs and exosomes released from OE-MSCs. **(A)** Immunophenotypes of OE-MSCs were detected by flow cytometry, including CD29, CD90, CD44, CD34, CD45, and CD11b. **(B)** Expression of vimentin and nestin (red) in OE-MSCs was detected by immunofluorescence with corresponding antibodies (original magnification ×200, bar = 30 μm). **(C)** OE-MSCs were cultured in osteogenic/adipocytes induction medium for 2/3 weeks, and the induced cells were then stained with oil red O/alizarin red, respectively (original magnification ×200, bar = 30 μm). **(D)** Western blot analysis was used to detect the CD9, CD63, and calnexin in OE-MSCs-Exos and OE-MSCs. **(E, F)** Representative SEM **(E)** (bar = 500nm) and TEM **(F)** (bar = 200 nm) micrographs of OE-MSCs-Exos. **(G)** Size profile of OE-MSCs-Exos was analyzed by NTA. Results are representative of three independent experiments.

OE-MSCs-Exos were isolated from the supernatants of OE-MSCs by ultracentrifugation method. Before the extraction of exosomes, the characteristics of OE-MSCs were identified. Several surface phenotypic markers were analyzed in OE-MSCs. As shown in **Figure 1A**, expression of CD29, CD90, and CD44 was positive on OE-MSCs, while CD34, CD45, and CD11b were negative. Furthermore, two neural stem cells-related markers, nestin and vimentin, were also expressed in OE-MSCs (**Figure 1B**). Likewise, OE-MSCs possessed the capacity to differentiate into adipocytes and osteocytes under indicated conditions (**Figure 1C**). All these data suggest that OE-MSCs display typical characteristics of MSCs and have been successfully purified.

Next, characteristics of exosomes were analyzed in OE-MSCs-Exos. Western blot analysis showed the isolated OE-MSCs-Exos displayed typical phenotypic features of Exos, they expressed CD63 and CD9, but not calnexin (**Figure 1D**). OE-MSCs-Exos exhibited a typical spheroidal shape surrounded by a double-layer membrane (**Figures 1E, F**), and the size distribution was also consistent with the exosomes (**Figure 1G**). Together, these data indicate the successful isolation and purification of OE-MSCs-Exos.

### OE-MSCs-Exos Display Potent Immunosuppressive Effect on CD4^+^T Cells

**Figure 2:**
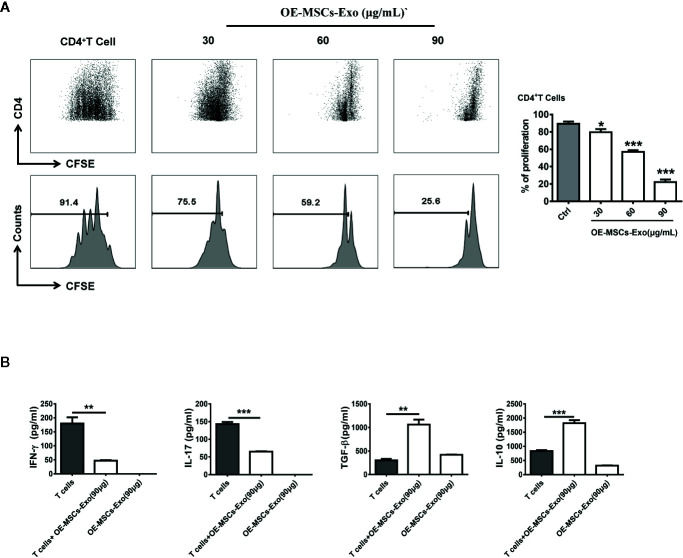
OE-MSCs-Exos suppress the CD4^+^T cell proliferation *in vitro*. **(A)** CFSE-labeled CD4^+^T cells were treated with OE-MSCs-Exos (30, 60, 90 μg/ml) under CD3 and CD28 stimulation. After 72 h, the T cell proliferation was determined by flow cytometric analysis of CFSE dilution. **(B)** Levels of IFN-γ, IL-17, TGF-β, and IL-10 were measured by ELISA in the supernatants of CD4^+^T cells with or without OE-MSCs-Exos (90 μg) and the lysis supernatant of OE-MSCs-Exos. Data are shown as mean ± SD from three independent experiments. ***p < 0.001, **p < 0.01, *p < 0.05.

To investigate whether OE-MSCs-Exos possess any immunosuppressive function, CD4^+^ T cells were stimulated with anti-CD3 mAb and anti-CD28 mAb in the presence of different concentrations of OE-MSCs-Exos. As shown in **Figure 2A**, the proliferation of CD4^+^T cells was inhibited in a dose-dependent manner. Moreover, levels of IFN-γ and IL-17 were reduced in the supernatant of OE-MSCs-Exos treated group, but the concentrations of several of suppressive cytokines, such as TGF-β and IL-10, were significantly increased (**Figure 2B**).

### Adoptive Transfer of OE-MSCs-Exos Attenuates the Severity of DSS-Induced Colitis

**Figure 3:**
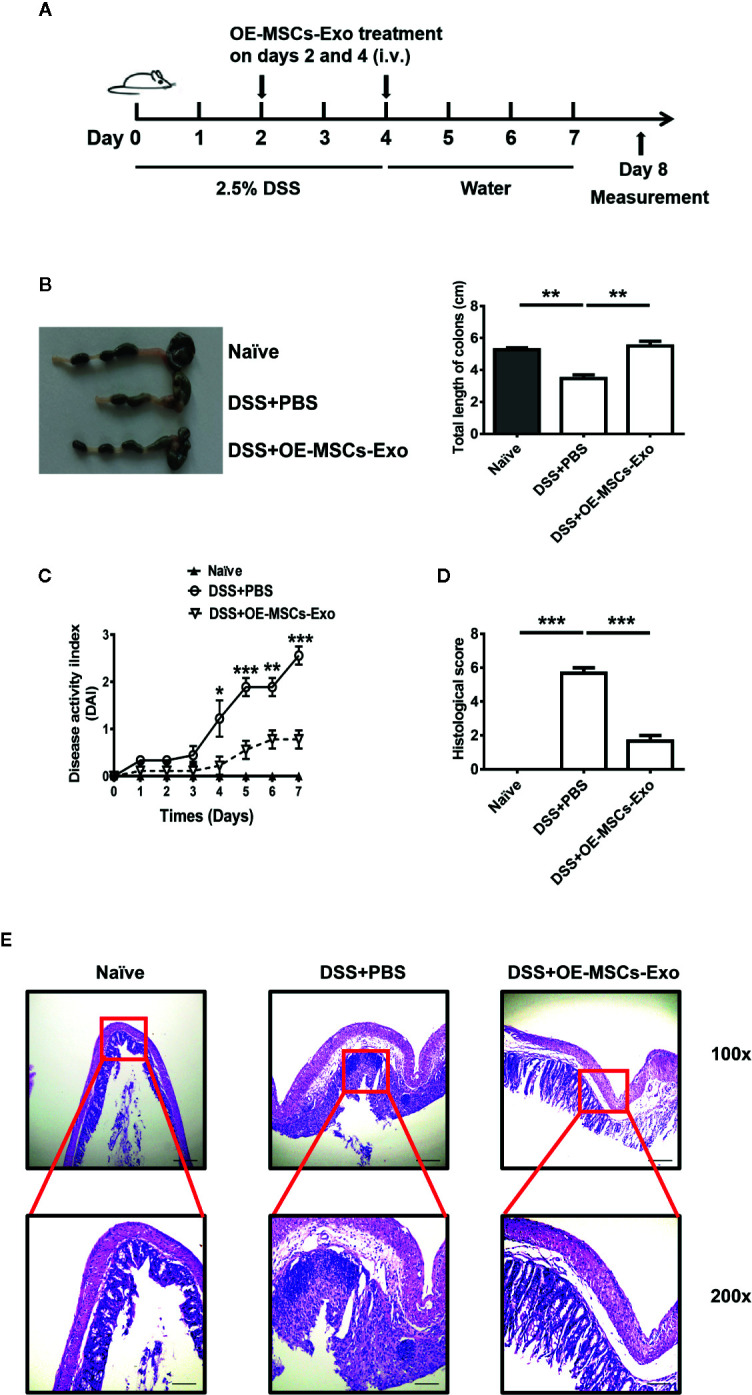
OE-MSCs-Exos protect against the murine experimental colitis. **(A)** Graphic scheme of DSS-induced colitis induction and OE-MSCs-Exos administration. C57BL/6 mice were subjected to 2.5% DSS in the drinking water for 4 days, followed by water treatment from day 4 to 7. OE-MSCs-Exos (60 μg/mouse) were i.v. injected into the mice on days 2 and 4 (n = 6/group). **(B)** Mice were sacrificed on day 8, the length of colons was measured. **(C)** DAI scores were evaluated and recorded everyday. **(D, E)** The histopathological detection of the colon sections was analyzed by H&E staining **(E)**, and the histopathological scores were determined **(D)**. Original magnification ×100 (upper, bar = 150 μm), ×200 (lower, bar = 50 μm). Data are shown as mean ± SD from three independent experiments. ***p < 0.001, **p < 0.01, *p < 0.05.

To evaluate the potential protective effect of OE-MSCs-Exos in DSS-induced IBD, OE-MSCs-Exos were administered to mice on days 2 and 4 after the colitis induction (**Figure 3A**). As shown in **Figure 3B**, the colons of the control group were obviously shortened and contracted when compared to the naïve mouse, whereas OE-MSCs-Exos treatment significantly increased the colon length and ameliorated the inflammation. Simultaneously, mice in the control group displayed remarkably elevated disease activity index (DAI). However, after the intravenous injection of OE-MSCs-Exos, the disease activity of mice was significantly decreased (**Figure 3C**). Furthermore, the histological examination showed that OE-MSCs-Exos treatment effectively retained the integral structure of the colon, with the reduced crypt loss, less lymphocytic infiltration, and lower histological scores when compared with the control group (**Figures 3D, E**).

### OE-MSCs-Exos Reduced Th1/Th17 Responses and Enhanced Treg Expansion in Murine Experimental Colitis

**Figure 4:**
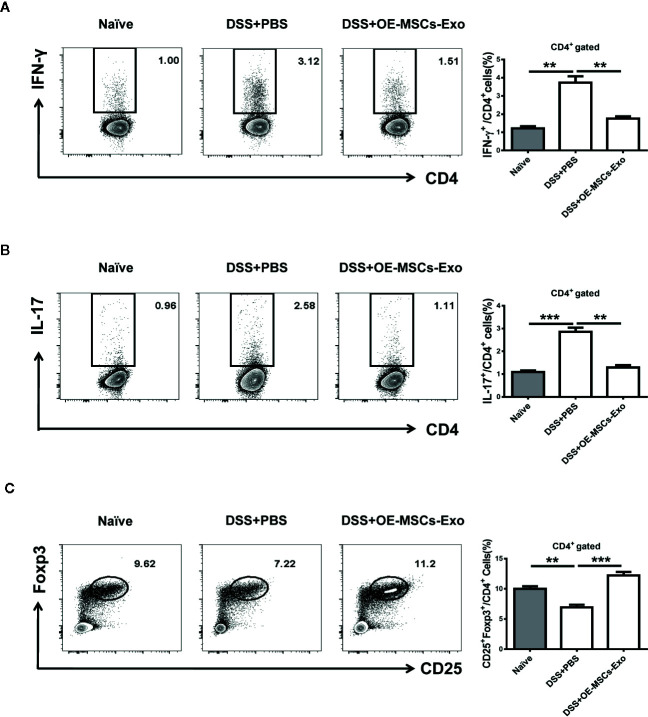
OE-MSCs-Exos reduce Th1/Th17 cells and expand Tregs in experimental colitis. C57BL/6 mice were subjected to 2.5% DSS in the drinking water for 4 days, followed by water treatment from day 4 to 7, and OE-MSCs-Exos (60 μg/mouse) were i.v. injected into the mice on days 2 and 4. Mice were sacrificed on day 8 (n = 6/group). **(A–C)** Proportions of Th1 **(A)**, Th17 **(B)**, and Treg **(C)** cells in MLNs were measured in each group by FCM. Data are shown as mean ± SD from three independent experiments. ***p < 0.001, **p < 0.01.

It has been acknowledged that Th1 and Th17 play important roles in the pathogenesis of IBD, and Treg cells exert potential anti-inflammatory function to protect against the development of IBD (26). Thereafter, the proportions of Th1, Th17, and Treg cells were analyzed in mice with experimental colitis from different groups. As shown in **Figures 4A, B**, the IBD mice displayed significant increased Th1 and Th17 cell responses when compared to the naïve mice. After the treatment of OE-MSCs-Exos, the percentages of Th1 and Th17 cells in mesenteric lymph nodes (MLNs) were remarkably reduced. Simultaneously, the frequency of Treg cells in MLNs was obviously enhanced after OE-MSCs-Exos administration (**Figure 4C**). Together, OE-MSCs-Exos can efficiently inhibit Th1 and Th17 cells and increase Treg cells in experimental colitis.

### OE-MSCs-Exos Inhibit the Differentiation of Th1/Th17 Cells but Promote the Treg Induction

**Figure 5:**
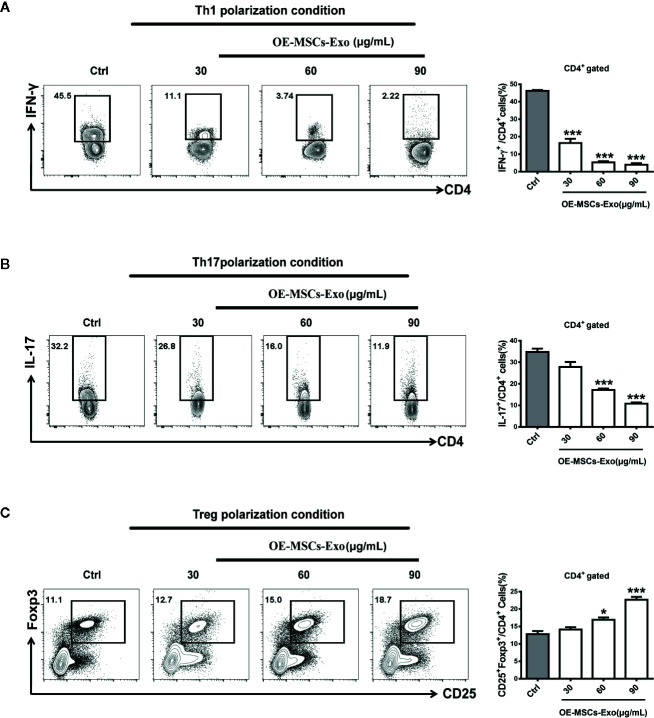
OE-MSCs-Exos suppress Th1 and Th17 induction while promote Treg differentiation. **(A–C)** 1×10^6^/ml naïve CD4^+^ T cells were purified from the spleen of C57BL/6 mice and cultured for 72 h in the presence of anti-CD3 and anti-CD28 mAbs under Th1 **(A)**, Th17 **(B)**, or Treg **(C)** differentiation conditions, respectively. Different concentrations of OE-MSCs-Exos (30, 60, 90 μg/ml) were added into each differentiation system. Frequencies of Th1, Th2, and Th17 cells were detected by FCM. Data are shown as mean ± SD from three independent experiments. ***p < 0.001, *p < 0.05.

Having observed the modulation of OE-MSCs-Exos in Th1, Th17, and Treg cells *in vivo*, we next established the differentiation system of Th1, Th17, and Treg *in vitro*, and evaluated whether OE-MSCs-Exos could affect the differentiation of these Th subsets directly, thus revealing the underlying mechanism of OE-MSCs-Exos in treating experimental colitis. As expected, under the Th1, Th17, and Treg differentiation conditions, OE-MSCs-Exos treatment could significantly inhibit the differentiation of Th1 and Th17 cells (**Figures 5A, B**), but promote the differentiation of Treg cells in a dose dependent manner (**Figure 5C**).

## Discussion

In this study, we first showed the typical MSCs phenotypic markers on OE-MSCs, and the capability of differentiating into osteocytes and adipocytes further confirmed they were of stem cell property. Moreover, the same as OE-MSCs, exosomes released by OE-MSCs were also demonstrated to possess immunosuppressive function, presented as the inhibition of CD4^+^ T cell proliferation, decreased IFN-γ, IL-17, and enhanced TGF-β, IL-10. Afterwards, adoptive transfer of OE-MSCs-Exos exhibited obvious therapeutic effect in the experimental colitis mice, both Th1 and Th17 cell responses were suppressed while Treg cells were expanded after OE-MSCs-Exos treatment. Furthermore, the *in vitro* data clarified that OE-MSCs-Exos could directly inhibit the differentiation of Th1/Th17 cells whereas promote the Treg induction. Taken together, OE-MSCs-Exos exerted their immunoregulation and effectively alleviated the disease severity in experimental colitis *via* regulating T cell responses.

MSCs have been reported to be found in various tissues, such as bone marrow, muscle, adipose, umbilical cord, placenta and so on (27, 28). Despite the sources are different, MSCs mostly share a similar characteristic phenotype. As we shown, OE-MSCs was a population of stem cells originate from olfactory lamina propria, possessing the typical characteristics of stem cells. Due to the immunosuppressive function of MSCs, they have been widely applicated in various autoimmune diseases and inflammatory diseases. Our previous data have demonstrated that OE-MSCs displayed stronger immunosuppressive capacity than BM-MSCs in regulating T cell responses (21). Recent years, exosomes released by MSCs have emerged as a novel and powerful secretory component of MSCs and aroused great interest. MSCs exert their immunologic properties mainly by paracrine pathway, and exosomes are supposed to be an essential manner in the process (29). Thus, the immunoregulation and anti‐inflammatory properties of MSCs are possibly found in MSC-derived exosomes. Indeed, several studies have identified the immunomodulatory properties of MSC-derived exosomes *in vitro* and animal models (16, 29, 30). In our study, exosomes derived from OE-MSCs were demonstrated to possess immunosuppressive function in regulating CD4^+^ T cell proliferation, and levels of inflammatory cytokines IL-17 and IFN-γ in OE-MSCs-Exos-treated supernatant were decreased, while the inhibitory cytokines TGF-β and IL-10 were increased, suggesting OE-MSCs-Exos might exert their immunoregulation function by suppressing the effector T cells and enhancing regulatory T cells.

MSC-Exos-based therapy is more than a compensation for MSC-based treatment. Exosomes have several potential advantages over the corresponding MSCs: first, the smaller size of exosomes can improve the therapeutic delivery to the desired sites by reducing the entrapment in capillaries after systemic administration. Also, the small size and less complicated structure are relatively easier for storage and production. Second, due to their acellular status, exosomes will have lower side effects and other risks induced by cells, such as less vascular obstructive propensity. In addition, the acellular status will protect exosomes from being modified by the *in vivo* environment, thus showing higher stability compared to cells. Besides, the properties of non-oncogenicity, immunosilence and tissue-specific homing allow MSC-Exos to be an ideal therapeutic tool in a variety of diseases (12, 29), including IBD. Although the etiology of IBD is unclear, dysregulated immune responses have been considered to be involved in the pathogenesis of IBD. In patients with IBD, percentages of effector T helper (Th) subsets, including Th1 and Th17 cell populations, were significantly increased in peripheral circulation. However, the proportion of Treg cells was showed to decrease (31, 32). The imbalance of effector Th cells and Tregs is supposed to be an essential element in the development of IBD. MSCs are known to regulate both innate and adaptive immune responses *via* secreting immunomodulatory factors and control the inflammation in IBD. As we previously reported, OE-MSCs possess several advantages when compared to BM-MSCs. Nasal lamina propria is the tissue easily to harvest and OE-MSCs can used for autologous transplantation. Furthermore, OE-MSCs exhibited higher proliferation profile and stronger suppressive capacity than BM-MSCs (20, 21, 33). Accumulating evidence have clarified exosomes can carry various mRNAs, microRNAs, and proteins from MSCs, and function as an extension of MSC’s biological role. Thus, considering the above advantages, exosomes released by OE-MSCs may serve as a potential cell-free therapy for IBD. As expected, adoptively transfer of OE-MSCs-Exos could efficiently ameliorate the severity of mice with experimental colitis, presented as reduced disease activity, less lymphocytic infiltration. Concurrently, Th1 and Th17 subpopulations were remarkably reduced whereas Treg cell increased in MLNs after OE-MSCs-Exos treatment. Mechanistically, OE-MSCs-Exos were demonstrated to inhibit the differentiation of Th1 and Th17 cells, but promote the induction of Treg cells *in vitro*. It has been acknowledged that MSCs-Exos can carry various regulatory molecules, such as IL-10, TGF-β, PGE2, IDO, PD-L1, and Gal-1 (34). However, the possible molecular mechanism for OE-MSCs-Exos to regulate the differentiation of T cell subsets still needs further exploration.

In summary, we have identified the immunoregulatory property of exosomes derived from OE-MSCs. Furthermore, OE-MSCs-Exos could exert their immunomodulation capacity to ameliorate disease severity in IBD mice, mainly by regulating Th-cell immune responses. These findings suggest that OE-MSCs-Exos represent a novel potential cell-free therapy for targeting inflammatory diseases.

## Data Availability Statement

The original contributions presented in the study are included in the article/supplementary material; further inquiries can be directed to the corresponding author.

## Ethics Statement

The animal study was reviewed and approved by Committee on the Use of Live Animals in Research and Teaching of Jiangsu University.

## Author Contributions

JT performed the experiments, analyzed the data, and wrote the paper. QZ, YZ, QB, and ZS performed the experiments. YH, HX, and KY analyzed the data. SW and KR designed the study and wrote the paper. All authors contributed to the article and approved the submitted version.

## Funding

This work was supported by the National Natural Science Foundation of China (Grant Nos. 81971542, 81701612), Natural Science Foundation of Jiangsu (Grant Nos. BK20170563, BK20190242), Summit of the Six Top Talents Program of Jiangsu Province (Grant No. 2017-YY-006), and Research Project of Jiangsu Commission of Health (Grant No. K2019019).

## Conflict of Interest

The authors declare that the research was conducted in the absence of any commercial or financial relationships that could be construed as a potential conflict of interest.
